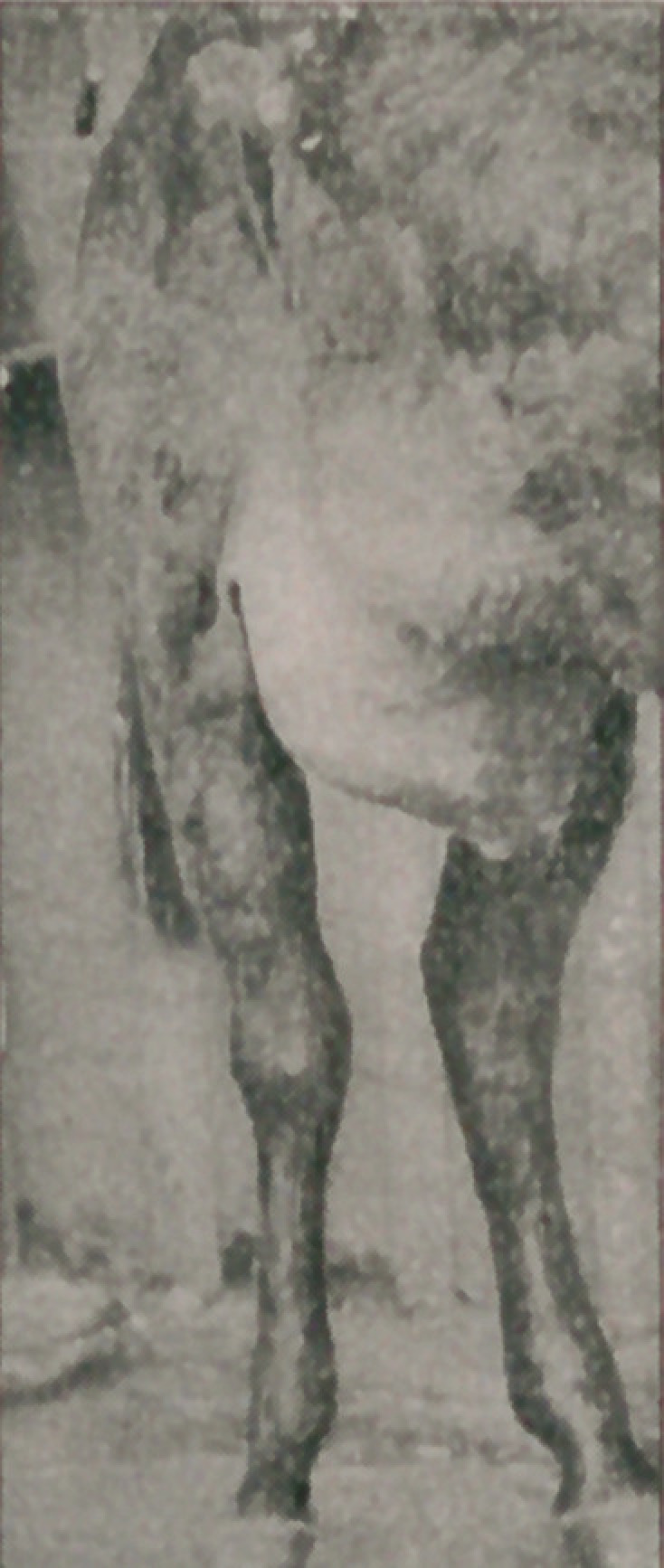# Traumatic Ventral Hernia

**Published:** 1896-04

**Authors:** 


### TRAUMATIC VENTRAL HERNIA.

THE above illustration is of a case coming under the observation of Dr. Joseph M. Good, of Chattanooga, Tenn.; the subject a mule, and the hernia of special interest, owing to its size and the fact that the animal lived for a period of fifteen days, when it was destroyed.

## Figures and Tables

**Figure f1:**
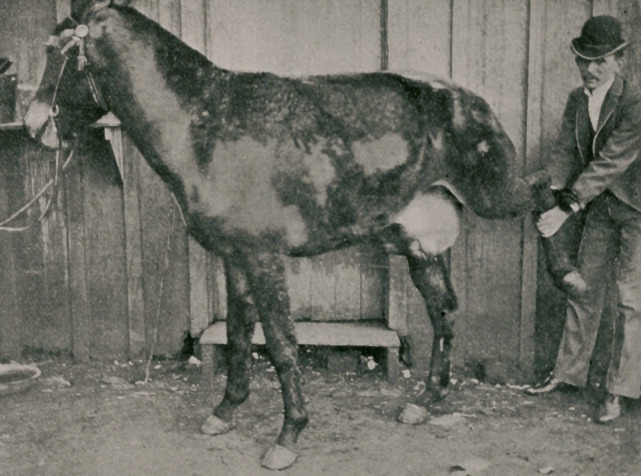


**Figure f2:**